# Fingolimod ameliorates chronic experimental autoimmune neuritis by modulating inflammatory cytokines and Akt/mTOR/NF‐κB signaling

**DOI:** 10.1002/brb3.2965

**Published:** 2023-03-14

**Authors:** Yuan Feng, Fang Feng, Shuyi Pan, Jiewen Zhang, Wei Li

**Affiliations:** ^1^ Department of Neurology Henan Provincial People's Hospital Zhengzhou China; ^2^ Department of Neurology Renmin Hospital of Wuhan University Wuhan China; ^3^ Department of Neurology The First Affiliated Hospital of Zhengzhou University Zhengzhou China; ^4^ Department of Hyperbaric Medicine 6th Medical Center of PLA General Hospital Beijing China

**Keywords:** chronic experimental autoimmune neuritis, chronic inflammatory demyelinating polyradiculoneuropathy, fingolimod, NF‐κB, peripheral neuropathy

## Abstract

**Objective:**

Chronic inflammatory demyelinating polyradiculoneuropathy (CIDP) is an immune‐mediated disease that targets the myelin sheaths of the peripheral nerves. Fingolimod is a sphingosine 1 phosphate (S1P) receptor antagonist with a high affinity for S1P receptors through the Akt–mTOR pathway, and prior research has suggested that it might be helpful in autoimmune illnesses.

**Methods:**

Chronic experimental autoimmune neuritis (c‐EAN) was induced by immunizing Lewis rats with the S‐palm P0(180–199) peptide, and then the treatment group was intraperitoneally injected with fingolimod (1 mg/kg) daily. Hematoxylin and eosin staining was used to assess the severity of nerve injury. Immunohistochemistry staining showed that fingolimod's anti‐inflammatory effects on c‐EAN rats might be realized through the NF‐κB signaling pathway. Tumor necrosis factor‐α (TNF‐α), interferon‐γ (INF‐γ), interleukin‐1beta (IL‐1β), interleukin 6 (IL‐6), inducible nitric oxide synthase (iNOS), and intercellular adhesion molecule‐1 (ICAM‐1) were measured to evaluate the inflammation levels, and pAkt, p‐S6, and p‐p65 were used to measure the abundance of downstream activation markers to determine whether the Akt/mTOR/NF‐κB signaling pathway was activated in the c‐EAN model.

**Results:**

Fingolimod treatment reduced the inflammatory reaction and the expression of NF‐κB in sciatic nerves. It also decreased the mRNA levels of the proinflammatory cytokines TNF‐α, IFN‐γ, IL‐1β, IL‐6, iNOS, and ICAM‐1 and pAkt, p‐S6, and p‐p65, representing the Akt/mTOR/NF‐κB signaling pathway.

**Conclusion:**

Our data showed that fingolimod could improve the disease course, alleviate the decrease in inflammation, and reduce proinflammatory cytokines through the Akt/mTOR/NF‐κB axis in c‐EAN rats, which could be beneficial for the development of CIDP‐related research.

## Graphical abstract

Our study showed that fingolimod could improve the disease course. Fingolimod alleviates the decrease in inflammation and reduces proinflammatory cytokines through the Akt/mTOR/NF‐kB axis in c‐EAN rats, which could be beneficial for the development of CIDP‐related research.

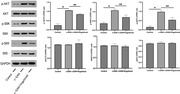

## 1 INTRODUCTION

Chronic inflammatory demyelinating polyradiculoneuropathy (CIDP) is an acquired immune‐mediated peripheral neuropathy characterized by sensorimotor disorder of the extremities with a relapsing–remitting or slowly progressive course and accompanied by weakened or eliminated tendon reflexes. The characteristics of CIDP are that the pathology of nerve demyelination and electrophysiology show slow conduction velocities and conduction blocks (Kuwabara & Misawa, 2019). CIDP is the most common chronic autoimmune neuropathy and treatable disease, so it should be considered first when a patient has progressive symmetric or asymmetric demyelinating polyneuropathy. The most widely used therapeutics for CIDP include intravenous immunoglobulin, corticosteroids, and plasma exchange, but the reaction to each immunotherapy is variable among different patients (Franques, 2019; Lehmann et al., 2019). It is unclear whether corticosteroids improve the impairment compared with no treatment, while because of their widespread availability, low cost, and low‐quality evidence in studies, they are usually used in the clinic. IVIg exerts anti‐inflammatory activity in autoimmune neuropathies, which includes Fc‐dependent and Fab‐dependent mechanisms, such as neutralization of autoantibodies, inhibition and abrogation of activated complement, alteration of FcR expression, and so forth. In addition, IVIg has fewer side effects and fewer adverse events than corticosteroids (Debs et al., 2017; Merkies et al., 2019). PE is a short‐term established treatment in several autoimmune diseases, including CIDP, which removes circulating autoantibodies and other immune factors, and as reported in several studies, it has the same efficacy as IVIg, but rapid deterioration can occur afterward (Chaigne & Mouthon, 2017; Lieker et al., 2017). Patients with CIDP treated with PE usually require other maintenance therapies also because it is inconvenient and high risk compared with medication, and it is not traditionally used in clinical practice. Currently, most patients are mainly treated with corticosteroids and immunoglobulin. At the same time, when the response to corticosteroids or IVIg is inadequate, other immunosuppressants, such as azathioprine, cyclophosphamide, or methotrexate, are used, but there have been no large‐scale clinical studies proving that they have a definite therapeutic effect (Bunschoten et al., 2019; Mahdi‐Rogers et al., 2017; Oaklander et al., 2017). In this study, Lewis rats were immunized with the P0(180−199) peptide thiopalmitoylated (S‐palm P0(180−199)) at cysteine 181 to create a chronic experimental autoimmune neuritis (c‐EAN) model, which had the highest similarity to CIDP and can be used for translational drug studies (Kremer et al., 2019).

Fingolimod is a receptor antagonist of sphingosine 1 phosphate (S1P), which has a high binding affinity for S1P receptors. As a new oral immunomodulator, its chemical structure and mechanism of action are both different from those of other immunosuppressants. Fingolimod was approved by the Food and Drug Administration, followed by the European Medicines Agency in 2011 for relapsing–remitting multiple sclerosis treatment (Brinkmann et al., 2010; Kappos et al., 2015; Wu et al., 2021). Previous studies have suggested that it also plays a beneficial role in some animal models of autoimmune diseases, including type 1 diabetes, lupus nephritis, systemic lupus erythematosus, and experimental autoimmune encephalomyelitis (EAE), by inhibiting macrophage and IL‐17+ cell infiltration in the peripheral nervous system (PNS), selectively confining lymphocytes in lymph nodes, reducing autoreactive T‐cell recruitment to the central nervous system, and negatively regulating proinflammatory signaling pathways (Cipriani et al., 2015; Cui et al., 2017; Hou et al., 2016; Shi et al., 2018; Tsuji et al., 2012; Yang et al., 2021).

A study of fingolimod in animal models of c‐EAN proved that it could inhibit macrophage and IL‐17+ cell infiltration in PNS. Oral fingolimod for CIDP in the clinic was studied in a double‐blind, multicenter, randomized controlled trial, which was terminated due to lack of efficacy (Hughes et al., 2018). There were explanations for the results of that study, such as that the study design was not suitable, or the patients were all in a stable stage, so fingolimod does not clearly play a role, or not all CIDP patients can benefit from fingolimod clinically. In our study, we investigated whether fingolimod is beneficial to c‐EAN rats and more deeply researched its mechanism for the regulation of inflammatory factors and signaling pathways.

## 2 MATERIALS AND METHODS

### 2.1 Animals

Male Lewis rats at 6−7 weeks of age and weighing 210−230 g were obtained from the Laboratory Animal Centre of Wuhan University (Wuhan, China). The rats were maintained in groups in a well‐ventilated room at 22 ± 2°C on a 12‐h light/1‐h dark cycle with free access to food and water. All experiments were approved by the Laboratory Animal Welfare and Ethical Committee of Wuhan University Renmin Hospital (Issue No. 20191111).

### 2.2 Induction of c‐EAN and clinical symptoms

Rats were randomly assigned to three groups (*n* = 10/group): the control group, the c‐EAN group, and the fingolimod treatment group. The control group rats were induced by subcutaneous injection into the rear tail vein of the rats with 200 μL of inoculum containing 0.5 mg of *Mycobacterium tuberculosis* (strain H37RA, Difco) emulsified in 100 μL of saline and 100 μL of incomplete Freund's adjuvant (IFA) (Sigma). The c‐EAN group rats were injected with 200 μL of inoculum, similar to the control group, but 200 μg of S‐palmitoylated P0(180−199) peptide was added. The S‐palmitoylated P0(180−199) peptide [AC(palm)KRGRQ TPVLYAMLDHSRS], obtained by thiopalmitoylation of residue cysteine at position 181, was synthesized by Hefei Guotai Biotechnology Co., Ltd. Then, the control group and the c‐EAN group were treated with normal saline. The fingolimod treatment group was the same as the c‐EAN rats, but they were injected intraperitoneally with fingolimod (1 mg/kg) every day from the first day of model creation to Day 31. This dosage has been shown to ameliorate EAN and c‐EAN in rats (Kremer et al., 2019; Zhang et al., 2008).

The clinical scores were assessed daily from Day 1 until 61 dpi (*n* = 5). The severity of clinical symptoms was scored as follows: 0 = normal; 1 = reduced tonus of tail; 2 = limp tail; impaired righting; 3 = absent righting; 4 = gait ataxia; 5 = mild paresis of the hind limbs; 6 = moderate paraparesis; 7 = severe paraparesis or paraplegia of the hind limbs; 8 = tetra paresis; 9 = moribund; and 10 = death.

### 2.3 Histological studies

Bilateral sciatic nerves were excised, and nerves were stained with hematoxylin and eosin (H&E) to evaluate overall histology and inflammation. The level of inflammation was quantified as follows: 0, no inflammation; 1, cellular infiltrates only tissue around the sciatic nerve; 2, mild cellular infiltrates; 3, moderate cellular infiltrates; and 4, serious cellular infiltrates (Han et al., 2013; Zhang et al., 2014).

### 2.4 Immunohistochemistry

Transcription factors related to inflammation in the sciatic nerve, including nuclear factor κB (NF‐κB), retinoic acid‐related orphan receptor alpha (RORα), and signal transducers and activators of transcription 5 (STAT5), were analyzed by immunohistochemical staining. The sciatic nerves were fixed and embedded in paraffin and then serially sectioned into 5‐μm‐thick sections, dewaxed, and washed three times with phosphate‐buffered saline (PBS) for 5 min each time. The sections were heated at 80°C for 10 min in EDTA antigen retrieval solution and washed three times with PBS for 5 min each time. Endogenous peroxidase was inhibited with 3% hydrogen peroxide solution for 10 min, washed three times with PBS for 5 min each time, and blocked with 5% bovine serum albumin for 20 min after drying. Sections were incubated overnight with the following monoclonal antibodies: anti‐NF‐κB (1:100; rabbit; Abcam), anti‐RORα (1:200; rabbit; Abcam), and anti‐Stat5 (1:100; rabbit; Abcam). Then, sections were washed three times with PBS for 5 min each time. The secondary antibody of the corresponding species (1:1500; HRP‐labeled goat anti‐rabbit; KPL) was added and incubated at 37°C for 50 min, followed by development with 3,3′‐diaminobenzidine substrate for antibodies. The slices were treated with 75%, 90%, 100%, and 100% gradient alcohol for 10 min, dehydrated, dried, cleared with xylene, and sealed with neutral gum. Counting was performed by two observers blinded to the therapy received using Fiji software.

### 2.5 Quantitative real‐time PCR analysis

Quantitation of the transcript levels of splenocyte cytokines was performed by quantitative real‐time PCR (qRT‐PCR). Tumor necrosis factor‐α (TNF‐α), interferon‐γ (INF‐γ), interleukin‐1beta (IL‐1β), interleukin 6 (IL‐6), inducible nitric oxide synthase (iNOS), and intercellular adhesion molecule‐1 (ICAM‐1) mRNA transcript levels were analyzed relative to the controls in splenocytes (*n* = 5). Total RNA from the spleen was extracted using TRIzol (Ambion) and treated with DNase 1, and RNA was reverse transcribed into cDNA using the RevertAid First Strand cDNA Synthesis Kit (Thermo) following the manufacturer's instructions. Real‐time PCR was performed with the following primers: TNF‐α (forward: 5′‐ACCTTATCTACTCCCAGGTTCT‐3′, reverse: 5′‐GGCTGACTTTCTCCTGGTATG‐3′), IFN‐γ (forward: 5′‐CGAATCGCACCTGATCACTAA‐3′, reverse: 5′‐TGGATCTGTGGGTTGTTCAC‐3′), IL‐1β (forward: 5′‐CTATGGCAACTGTCCCTGAA‐3′, reverse: 5′‐GGCTTGGAAGCAATCCTTAATC‐3′), IL‐6 (forward: 5′‐ GAAGTTAGAGTCACAGAAGGAGTG‐3′, reverse: 5′‐ GTTTGCCGAGTAGACCTCATAG‐3′), iNOS (forward: 5′‐TGGAGCGAGTTGTGGATTG‐3′, reverse: 5′‐ CCTCTTGTCTTTGACCCAGTAG‐3′), and ICAM‐1 (forward: 5′‐GTATCCATCCATCCCACAGAAG‐3′, reverse: 5′‐CAGTTGTGTCCACTCGATAGTT‐3′). SYBR Green PCR Mix (Takara) was used for quantitative assays with a 7500 Real‐Time RCR System (Applied Biosystems, USA). Relative TNF‐α, IFN‐γ, IL‐1β, IL‐6, iNOS, and ICAM‐1 mRNA levels were calculated using the 2^−ΔΔCT^ method.

### 2.6 Western blot analysis

Sciatic nerves were dissected (*n* = 5) and frozen immediately in liquid nitrogen. Total protein was extracted, quantified using a bicinchoninic acid (BCA) protein assay reagent kit (ASPEN), and heated for 10 min at 95°C. Equal amounts of proteins were separated by 4%–12% Bis–Tris sodium dodecyl sulfate‒polyacrylamide gel electrophoresis (Baiqiandu) and transferred to polyvinylidene fluoride membranes (Millipore). Nonspecific binding sites were blocked by incubation in 5% nonfat dry milk in TBST (Tris‐buffered saline, 0.1% Tween 20) for 1 h. Membranes were then incubated at 4°C for 12 h with primary anti‐Akt antibodies (1:2000; Cell Signaling Technology), anti‐phospho‐Akt (pAkt; 1:2000; Cell Signaling Technology), anti‐s6k (1:1000; Cell Signaling Technology), anti‐phospho‐s6k (ps6k; 1:1000; Cell Signaling Technology), anti‐p65 (1:1000; Abcam), anti‐phospho‐p65 (1:1000; Abcam), or control GAPDH (1:6000, Abcam). After three washes in TBST, bound antibodies were detected by incubation with the corresponding secondary antibodies (1:50,000) for 1 h. Data were analyzed using Image‐Pro Plus software, version 6.3 (Media Cybernetics, Inc., Rockville, MD, USA).

### 2.7 Statistical analysis

Data were analyzed using SPSS software (version 21.0). The data are expressed as the mean ± standard deviation. Significant differences in clinical scores between pairs of groups were examined using repeated measures analysis of variance when the data were normally distributed, and the Mann–Whitney *U* test was used when the variable was not normally distributed. Comparisons among groups were performed by one‐way ANOVA followed by Bonferroni's correction method (or the Kruskal–Wallis test for nonparametric data). Significance levels were set at *p* < .05.

## 3 RESULTS

### 3.1 Fingolimod reduces the clinical symptoms of c‐EAN

**FIGURE 1:**
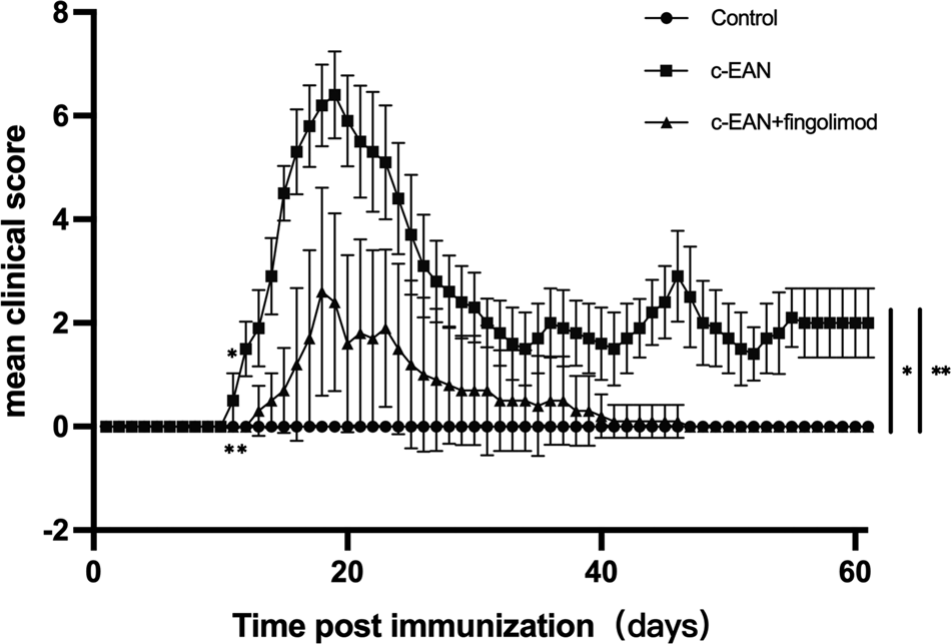
Fingolimod reduced the severity of chronic experimental autoimmune neuritis (c‐EAN). From the 11th day, compared with the control group and the fingolimod treatment group, the clinical score of the model group was significantly higher, and the difference was statistically significant. At the end of the experiment, the rats in the c‐EAN group still had some disabilities, which was statistically significant compared with the control group and the fingolimod treatment group. Data are expressed as the mean ± SD of the clinical scores (*n* = 5, *p ＜0.05 vs. the control group; **p < 0.05 vs. the c‐EAN group).

We examined the effect of fingolimod on c‐EAN rats. Animals were randomly divided into a control group, a c‐EAN group, and a fingolimod treatment group. They were treated as mentioned above. Then, the control group and c‐EAN group were treated with normal saline, and the fingolimod treatment group was intraperitoneally treated with fingolimod (1 mg/kg) once daily for 30 days from the first day postimmunization. The c‐EAN group exhibited stiffness and difficulty walking from the 11th day, and symptoms continued to worsen until 19 days postimmunization (dpi), with a maximal clinical score of 6.4 ± 0.84. The disability persisted until 60 dpi without complete remission. Symptoms occurred in the fingolimod treatment group from 13 dpi and became most serious at 18 dpi with a score of 2.6 ± 2.0. Compared with the c‐EAN group, the fingolimod treatment group exhibited decreased maximal clinical scores and faster recovery (Figure 1).

### 3.2 Fingolimod improves the damage of the pathology in a c‐EAN rat

**FIGURE 2:**
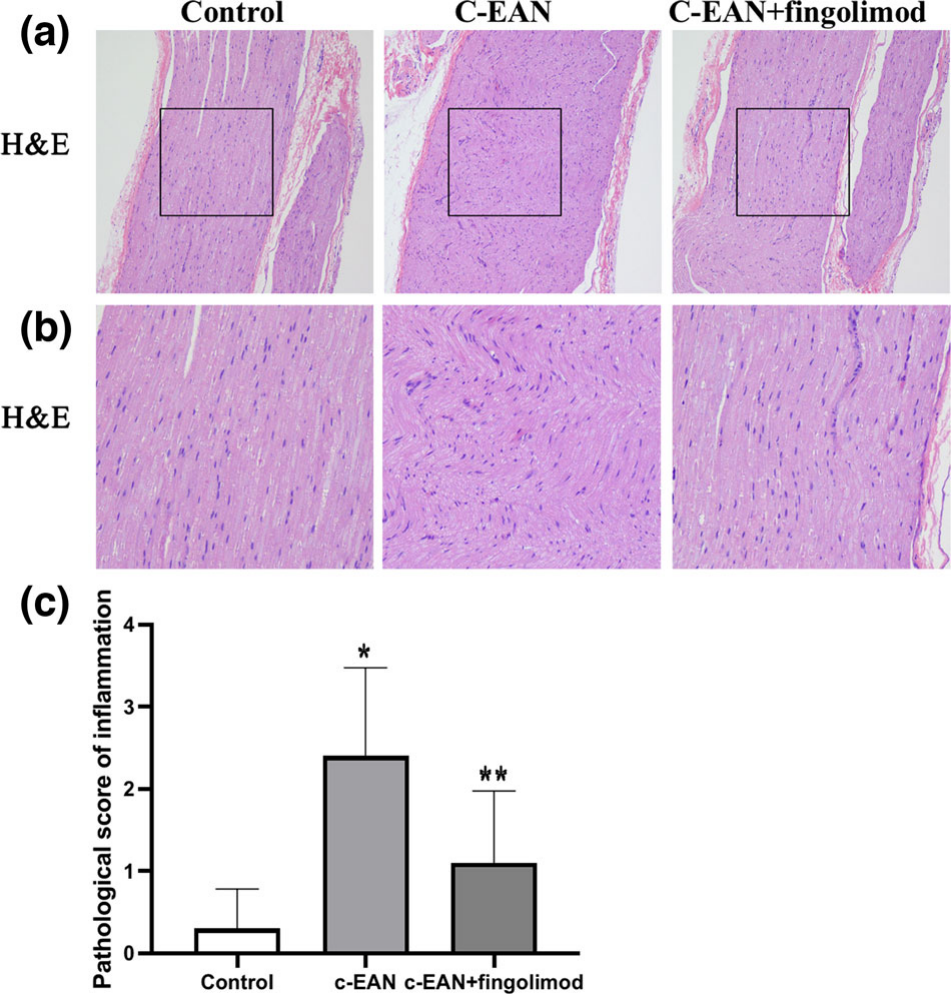
Fingolimod treatment reduced inflammatory infiltration in the c‐EAN rat. The pathological scores of inflammation in the different groups were expressed as mean ± SD. (A) The hematoxylin and eosin (H&E) staining of a longitudinal section of the sciatic nerve (H&Ex100). (B) High‐magnification image of area indicated by the box in panel (A). (C) The quantification of demyelination in the sciatic nerve of the rat (*n* = 5, *p ＜0.05 vs. the control group; **p ＜0.05 vs. the c‐EAN group).

We excised the sciatic nerves to be stained with H&E to evaluate inflammatory infiltration in rats. Compared with the control group, the sciatic nerves of the c‐EAN group and the fingolimod treatment group both appeared damaged by inflammatory infiltration, and damage in the fingolimod treatment groups was lighter than that in the c‐EAN rats, indicating that fingolimod improved the lesion of the pathology (Figure 2).

### 3.3 Fingolimod reduces the expression of NF‐κB in the sciatic nerve of rats

**FIGURE 3:**
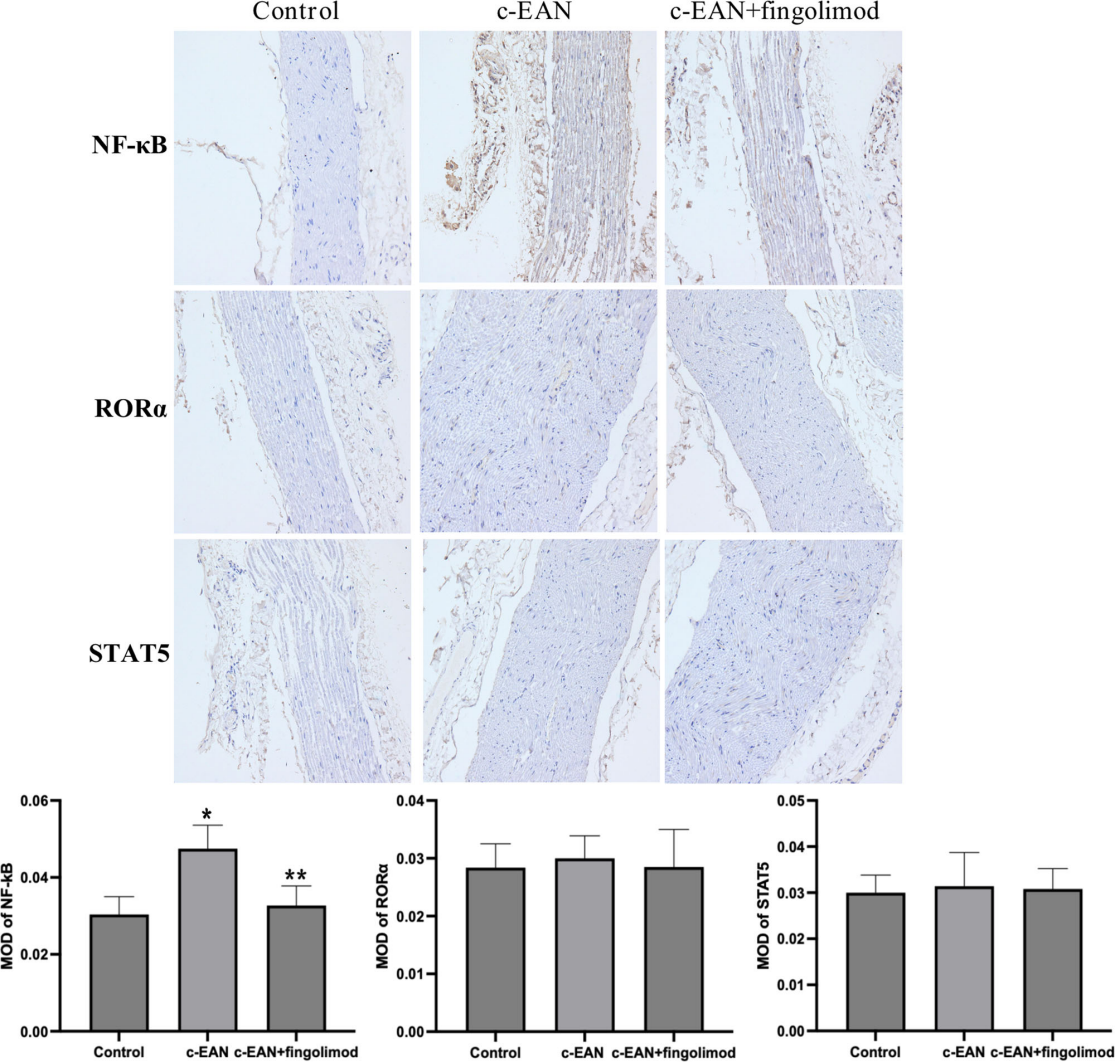
Fingolimod inhibits the expression of transcription factor NF‐κB infiltration in sciatic nerves of c‐EAN rats. Sciatic nerves sampled in the three groups of rats were labeled with NF‐κB, RORα, and STAT5 by immunohistochemical staining and the NF‐κB changes significantly in c‐EAN group and fingolimod treatment group (*n* = 5, *p ＜0.05 vs. the control group; **p ＜0.05 vs. the c‐EAN group).

NF‐κB, RORα, and STAT5 are all immune‐related transcription factors, and they were measured by immunohistochemical staining to determine whether they participated in the inflammatory changes in the sciatic nerves in the rats. Compared with the control group, the expression of NF‐κB in the sciatic nerves of C‐EAN rats increased significantly, but that of RORα and STAT5 did not change significantly. Compared with the c‐EAN group, the fingolimod treatment group showed a significant decrease in the expression of NF‐κB, while that of RORα and STAT5 showed no marked change. It can be seen from the above that fingolimod significantly reduced the expression of NF‐κB in the sciatic nerve but had little effect on that of RORα and STAT5, indicating that the improvement of the local anti‐inflammatory effect of fingolimod could be achieved by inhibiting NF‐κB signaling (Figure 3).

### 3.4 Fingolimod changed the inflammatory cytokines in c‐EAN rats

**FIGURE 4:**
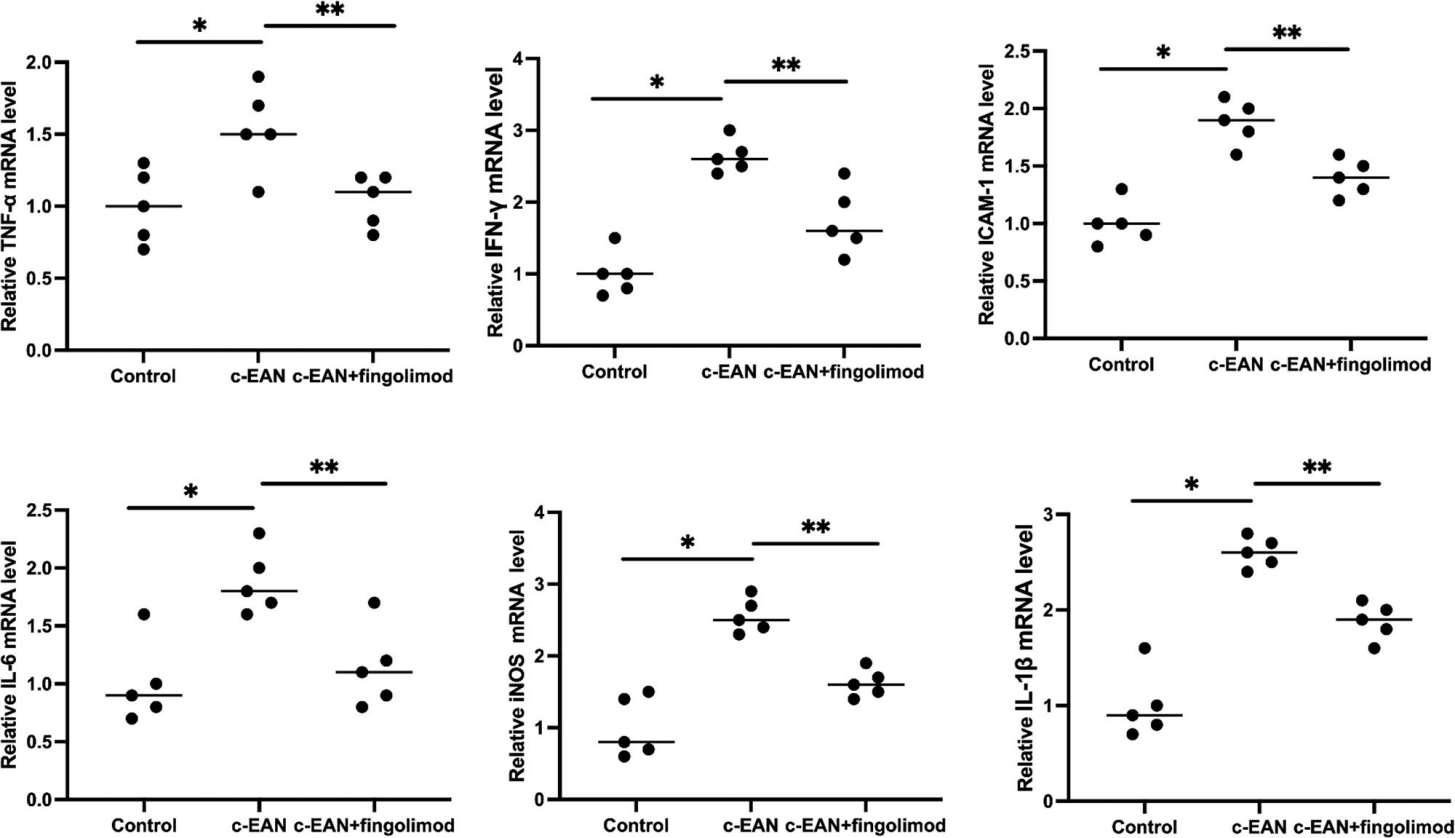
Fingolimod decreases the level of proinflammation cytokine in the spleen. The mRNA levels of TNF‐α, INF‐γ, IL‐1β, IL‐6, iNOS, and ICAM‐1 in spleens were analyzed by quantitative real‐time PCR (qRT‐PCR) (*n* = 5, *p ＜0.05 vs. the control group; **p ＜0.05 vs. the c‐EAN group).

We researched the spleen's inflammatory cytokines to determine the fingolimod treatment effect on c‐EAN rats. The levels of TNF‐α, INF‐γ, IL‐1β, IL‐6, iNOS, and ICAM‐1 were measured in the three groups at 19 dpi for evaluation. The results showed that, compared with the control group, these proinflammatory factors in the c‐EAN group were significantly increased, and they were all decreased compared with the c‐EAN group (Figure 4).

### 3.5 Fingolimod suppresses the Akt/mTOR/NF‐κB pathway

**FIGURE 5:**
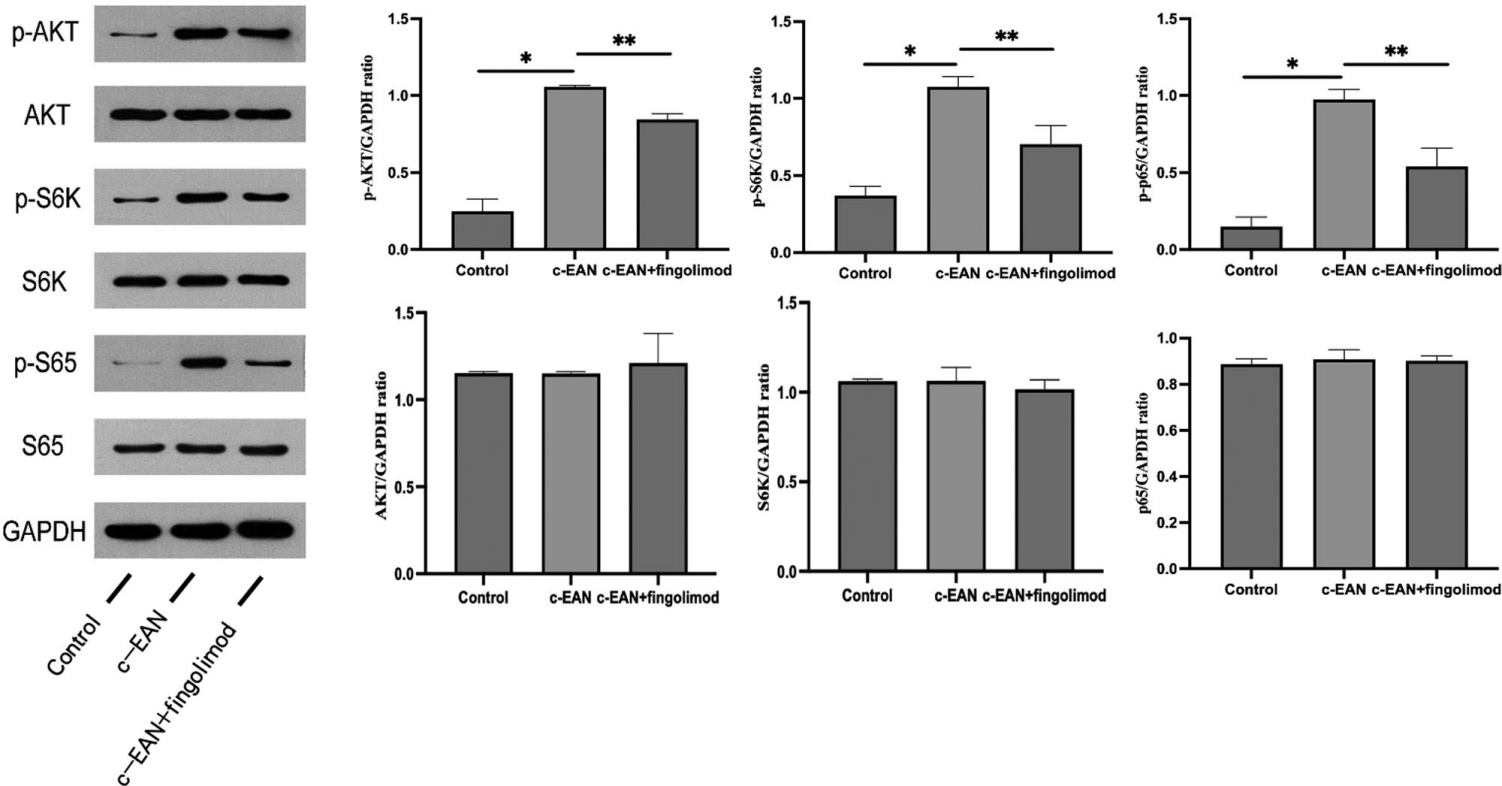
Fingolimod suppresses the Akt/mTOR/NF‐κB pathway. Representative western blot showing expression of the indicated proteins in control, EAN, and EAN + fingolimod cohorts. Relative levels of Akt, s6k, and s65 proteins were measured as a ratio of phosphorylated/unphosphorylated protein (*n* = 5, *p ＜0.05 vs. the control group; **p ＜0.05 vs. the c‐EAN group).

Phosphorylation of the kinases Akt, s6k, and p65 indicates that the Akt/mTOR/NF‐κB pathway is activated. We examined the expression of phospho‐Akt (p‐Akt), phospho‐s6k (p‐s6k), and phospho‐p65 (p‐p65) in the sciatic nerves of rats by western blotting (Figure 5). Compared with the control group, the expression of p‐Akt, p‐s6k, and p‐p65 was increased in the c‐EAN group. The onset of the c‐EAN model could activate the Akt/mTOR/NF‐κB pathway. Then, we measured p‐Akt, p‐s6k, and p‐p65 in the fingolimod treatment group, and they were all reduced in the sciatic nerves. This outcome indicates that fingolimod can alleviate the inflammatory response by regulating the Akt/mTOR/NF‐κB signaling pathway.

## 4 DISCUSSION

To date, there are few effective treatments for CIDP patients. Worse, some patients do not react to corticosteroids, IVIg, and PE, so they will always have physical disabilities, such as motor and sensory abnormalities. It is necessary for us to seek new therapeutic drugs that offer high efficacy. Fingolimod is a receptor antagonist of S1P1, which has a high binding affinity for S1P receptors, which mediate a wide range of biological functions and are expressed throughout the body. The mechanism of action of fingolimod is different from that of other immunosuppressants that are used to treat CIDP in the clinic. Previous studies have indicated that fingolimod treats cancer by inhibiting lymphocyte egress from secondary lymphoid tissues and the thymus and inducing lymphopenia (White et al., 2016). In addition, it plays a role in kidney transplantation, heart failure, and arrhythmia (Cannavo et al., 2017). It also has certain neuroprotective effects against intracerebral hemorrhage, amyotrophic lateral sclerosis, Alzheimer's disease, experimental autoimmune neuritis, and ischemic stroke, but it aggravated brain edema in the acute stage of cerebral ischemia in diabetic mice (Carreras et al., 2019; Egom et al., 2011; Fu et al., 2014; Kremer et al., 2019; Li et al., 2020; McGinley & Cohen, 2021; Skerjanec et al., 2005; Zhu et al., 2015). However, the mechanism of fingolimod in treating autoimmune diseases remains indeterminate.

In this study, we researched the therapeutic effects of fingolimod in c‐EAN rats, a rat model mimicking CIDP, and we found a new treatment strategy for patients. Our research showed that fingolimod was beneficial for c‐EAN rats. Fingolimod could improve the clinical symptoms of c‐EAN rats, reduce the maximum clinical score, expedite recovery, and shorten the course of the disease, in agreement with reported studies of EAN and EAE models. However, the effect of fingolimod on autoimmune neuropathy is not all effective, and contradictory results with ours have been reported in a mouse model of spontaneous autoimmune peripheral polyneuropathy (Huehnchen et al., 2018). A different result could have occurred because, in the last study, treatment was started when the clinical symptoms worsened and not at the beginning.

Fingolimod alleviated the inflammatory reaction in c‐EAN rats. Histopathology, H&E staining, molecular biology, and qRT‐PCR showed that fingolimod treatment could significantly reduce the severity of local and systemic inflammation in c‐EAN rats. The results of immunohistochemistry showed that the anti‐inflammatory effect of fingolimod on c‐EAN rats might be realized through the NF‐κB signaling pathway. TNF‐α is upstream of NF‐κB signaling, which can regulate it, and it plays an essential role in complex inflammatory responses in different manners (Mohammad Jafari et al., 2021; Ye et al., 2021). Studies have shown that fingolimod can attenuate the phosphorylation level of molecules in the NF‐κB pathway and decrease the inflammatory cytokines L‐1β and IL‐6 to improve neural defects and reduce neuronal damage in cerebral ischemia/reperfusion rats (Zhang et al., 2022). In addition, fingolimod treatment significantly reduced the expression of ICAM‐1 and INF‐γ in an experimental acute hemorrhage model (Rolland et al., 2013). These results were all consistent with our study, and they indicated that cytokines related to the NF‐κB signaling pathway could be regulated by fingolimod. Furthermore, the anti‐inflammatory effect of fingolimod also showed that it polarizes proinflammatory M1 macrophages toward anti‐inflammatory M2 macrophages and reduces chronic inflammation and tissue damage (Zhao et al., 2018).

The mammalian target of the rapamycin (mTOR) signaling pathway plays important roles in cell metabolism, growth, and proliferation, and its dysregulation has been implicated in changes in pathology. S1P1 is a receptor located upstream of the Akt/mTOR signaling pathway (Ishitsuka et al., 2014; Liu et al., 2009, 2010). Fingolimod, as a modulator of the S1P1 receptor, has an inhibitory effect on the inflammatory response by regulating the Akt/mTOR/NF‐κB pathway. Akt phosphorylation can activate the inflammation‐related transcription factor NF‐κB. NF‐κB exists in a variety of cells and can regulate immunity, inflammation, stress response, and so forth. It is the intersection of multiple inflammation‐related signaling pathways and can promote the occurrence of reactions and the release of proinflammatory cytokines. The phosphorylation of Akt, s6k, and p65 reflects the activity of this signaling pathway. In our study, we observed that the expression of p‐Akt, p‐s6k, and p‐p65 increased in the c‐EAN group, and these levels decreased in the fingolimod group, indicating that the Akt/mTOR/NF‐κB pathway was activated in the c‐EAN model and that fingolimod inhibited it. Recently, this pathway was also reported in a study of EAE mice treated with fingolimod (Müller et al., 2017). These results helped us to define the mechanism of fingolimod‐mediated immunoregulation in c‐EAN.

In our study, we found that fingolimod alleviated the inflammatory reaction at the sciatic nerve and regulated the expression of systemic inflammatory factors and signaling pathways. These results were consistent with previous reports in rats with autoimmune diseases. Of course, because of the experimental conditions, our research has certain limitations, such as the sample size and technical level. Several CIDP patients show little response to the therapeutic drugs that are used for CIDP patients in the clinic, and these drugs all have different receptors and mechanisms with fingolimod. On the one hand, we need more in‐depth research on the therapeutic mechanism of fingolimod in c‐EAN rats; on the other hand, more work must be done to determine whether fingolimod could be beneficial to CIDP patients in the clinic. At the same time, considering the adverse reactions to fingolimod, we require more research to seek the most suitable dose with minimal side effects.

## 5 CONCLUSIONS

In conclusion, this study showed that fingolimod ameliorated disease in c‐EAN rats, as evaluated by clinical scores, histopathology, and molecular biology. Moreover, we observed that fingolimod modulated inflammatory cytokines and affected the Akt/mTOR/NF‐κB axis in c‐EAN rats, which could be beneficial for CIDP patients.

## AUTHOR CONTRIBUTIONS

Yuan Feng performed the experimental research, read a lot of literature, and wrote the first draft. Shuyi Pan and Jiewen Zhang had the idea of performing this research, performed the literature search, and critically appraised the draft. Fang Feng fully assisted in the experimental procedure. Wei Li critically modified the grammar and participated in the statistical process. All authors read and approved the final manuscript.

## CONFLICT OF INTEREST STATEMENT

The authors declare no conflicts of interest.

### PEER REVIEW

The peer review history for this article is available at https://publons.com/publon/10.1002/brb3.2965

## Data Availability

The data sets generated and/or analyzed during the current study are available from the corresponding author on reasonable request.

## References

[brb32965-bib-0001] Brinkmann, V. , Billich, A. , Baumruker, T. , Heining, P. , Schmouder, R. , Francis, G. , & Burtin, P. (2010). Fingolimod (FTY720): Discovery and development of an oral drug to treat multiple sclerosis. Nature Reviews Drug Discovery, 9(11), 883–897. 10.1038/nrd3248 21031003

[brb32965-bib-0002] Bunschoten, C. , Jacobs, B. C. , Van den Bergh, P. Y. K. , Cornblath, D. R. , & van Doorn, P. A. (2019). Progress in diagnosis and treatment of chronic inflammatory demyelinating polyradiculoneuropathy. Lancet Neurology, 18(8), 784–794. 10.1016/s1474-4422(19)30144-9 31076244

[brb32965-bib-0003] Cannavo, A. , Liccardo, D. , Komici, K. , Corbi, G. , de Lucia, C. , Femminella, G. D. , & Rengo, G. (2017). Sphingosine kinases and sphingosine 1‐phosphate receptors: Signaling and actions in the cardiovascular system. Frontiers in Pharmacology, 8, 556. 10.3389/fphar.2017.00556 28878674 PMC5572949

[brb32965-bib-0004] Carreras, I. , Aytan, N. , Choi, J. K. , Tognoni, C. M. , Kowall, N. W. , Jenkins, B. G. , & Dedeoglu, A. (2019). Dual dose‐dependent effects of fingolimod in a mouse model of Alzheimer's disease. Scientific Reports, 9(1), 10972. 10.1038/s41598-019-47287-1 31358793 PMC6662857

[brb32965-bib-0005] Chaigne, B. , & Mouthon, L. (2017). Mechanisms of action of intravenous immunoglobulin. Transfusion and Apheresis Science, 56(1), 45–49. 10.1016/j.transci.2016.12.017 28161150

[brb32965-bib-0006] Cipriani, R. , Chara, J. C. , Rodríguez‐Antigüedad, A. , & Matute, C. (2015). FTY720 attenuates excitotoxicity and neuroinflammation. Journal of Neuroinflammation, 12, 86. 10.1186/s12974-015-0308-6 25953296 PMC4429813

[brb32965-bib-0007] Cui, K. , Ruan, Y. , Wang, T. , Rao, K. , Chen, Z. , Wang, S. , & Liu, J. (2017). FTY720 supplementation partially improves erectile dysfunction in rats with streptozotocin‐induced type 1 diabetes through inhibition of endothelial dysfunction and corporal fibrosis. Journal of Sexual Medicine, 14(3), 323–335. 10.1016/j.jsxm.2017.01.006 28162947

[brb32965-bib-0008] Debs, R. , Reach, P. , Cret, C. , Demeret, S. , Saheb, S. , Maisonobe, T. , & Viala, K. (2017). A new treatment regimen with high‐dose and fractioned immunoglobulin in a special subgroup of severe and dependent CIDP patients. International Journal of Neuroscience, 127(10), 864–872. 10.1080/00207454.2016.1269328 27918219

[brb32965-bib-0009] Egom, E. E. , Mohamed, T. M. , Mamas, M. A. , Shi, Y. , Liu, W. , Chirico, D. , & Lei, M. (2011). Activation of Pak1/Akt/eNOS signaling following sphingosine‐1‐phosphate release as part of a mechanism protecting cardiomyocytes against ischemic cell injury. American Journal of Physiology ‐ Heart and Circulatory Physiology, 301(4), H1487–1495. 10.1152/ajpheart.01003.2010 21705677 PMC3197364

[brb32965-bib-0010] Franques, J. (2019). Chronic inflammatory demyelinating polyneuropathy: Diagnosis and therapeutic update. Revue de Médecine Interne, 40(12), 808–815. 10.1016/j.revmed.2019.07.007 31677862

[brb32965-bib-0011] Fu, Y. , Hao, J. , Zhang, N. , Ren, L. , Sun, N. , Li, Y. J. , & Shi, F. D. (2014). Fingolimod for the treatment of intracerebral hemorrhage: A 2‐arm proof‐of‐concept study. JAMA Neurology, 71(9), 1092–1101. 10.1001/jamaneurol.2014.1065 25003359

[brb32965-bib-0012] Han, S. , Zhang, F. , Hu, Z. , Sun, Y. , Yang, J. , Davies, H. , & Fang, M. (2013). Dose‐dependent anti‐inflammatory and neuroprotective effects of an ανβ3 integrin‐binding peptide. Mediators of Inflammation, 2013, 268486. 10.1155/2013/268486 24347822 PMC3855988

[brb32965-bib-0013] Hou, H. , Cao, R. , Miao, J. , Sun, Y. , Liu, X. , Song, X. , & Guo, L. (2016). Fingolimod ameliorates the development of experimental autoimmune encephalomyelitis by inhibiting Akt‐mTOR axis in mice. International Immunopharmacology, 30, 171–178. 10.1016/j.intimp.2015.11.024 26632437

[brb32965-bib-0014] Huehnchen, P. , Boehmerle, W. , & Endres, M. (2018). Fingolimod therapy is not effective in a mouse model of spontaneous autoimmune peripheral polyneuropathy. Scientific Reports, 8(1), 5648. 10.1038/s41598-018-23949-4 29618748 PMC5884804

[brb32965-bib-0015] Hughes, R. , Dalakas, M. C. , Merkies, I. , Latov, N. , Léger, J. M. , Nobile‐Orazio, E. , & Hartung, H. P. (2018). Oral fingolimod for chronic inflammatory demyelinating polyradiculoneuropathy (FORCIDP Trial): A double‐blind, multicentre, randomised controlled trial. Lancet Neurology, 17(8), 689–698. 10.1016/s1474-4422(18)30202-3 30001923

[brb32965-bib-0016] Ishitsuka, A. , Fujine, E. , Mizutani, Y. , Tawada, C. , Kanoh, H. , Banno, Y. , & Seishima, M. (2014). FTY720 and cisplatin synergistically induce the death of cisplatin‐resistant melanoma cells through the downregulation of the PI3K pathway and the decrease in epidermal growth factor receptor expression. International Journal of Molecular Medicine, 34(4), 1169–1174. 10.3892/ijmm.2014.1882 25109763

[brb32965-bib-0017] Kappos, L. , O'Connor, P. , Radue, E. W. , Polman, C. , Hohlfeld, R. , Selmaj, K. , & Francis, G. (2015). Long‐term effects of fingolimod in multiple sclerosis: The randomized FREEDOMS extension trial. Neurology, 84(15), 1582–1591. 10.1212/wnl.0000000000001462 25795646 PMC4408283

[brb32965-bib-0018] Kremer, L. , Taleb, O. , Boehm, N. , Mensah‐Nyagan, A. G. , Trifilieff, E. , de Seze, J. , & Brun, S. (2019). FTY720 controls disease severity and attenuates sciatic nerve damage in chronic experimental autoimmune neuritis. Journal of Neuroinflammation, 16(1), 54. 10.1186/s12974-019-1441-4 30825874 PMC6397476

[brb32965-bib-0019] Kuwabara, S. , & Misawa, S. (2019). Chronic inflammatory demyelinating polyneuropathy. Advances in Experimental Medicine and Biology, 1190, 333–343. 10.1007/978-981-32-9636-7_21 31760654

[brb32965-bib-0020] Lehmann, H. C. , Burke, D. , & Kuwabara, S. (2019). Chronic inflammatory demyelinating polyneuropathy: Update on diagnosis, immunopathogenesis and treatment. Journal of Neurology, Neurosurgery, and Psychiatry, 90(9), 981–987. 10.1136/jnnp-2019-320314 30992333

[brb32965-bib-0021] Li, W. , He, T. , Jiang, L. , Shi, R. , Song, Y. , Mamtilahun, M. , & Wang, Y. (2020). Fingolimod inhibits inflammation but exacerbates brain edema in the acute phases of cerebral ischemia in diabetic mice. Frontiers in Neuroscience, 14, 842. 10.3389/fnins.2020.00842 32848587 PMC7432267

[brb32965-bib-0022] Lieker, I. , Slowinski, T. , Harms, L. , Hahn, K. , & Klehmet, J. (2017). A prospective study comparing tryptophan immunoadsorption with therapeutic plasma exchange for the treatment of chronic inflammatory demyelinating polyneuropathy. Journal of Clinical Apheresis, 32(6), 486–493. 10.1002/jca.21546 28485075

[brb32965-bib-0023] Liu, G. , Burns, S. , Huang, G. , Boyd, K. , Proia, R. L. , Flavell, R. A. , & Chi, H. (2009). The receptor S1P1 overrides regulatory T cell‐mediated immune suppression through Akt‐mTOR. Nature Immunology, 10(7), 769–777. 10.1038/ni.1743 19483717 PMC2732340

[brb32965-bib-0024] Liu, G. , Yang, K. , Burns, S. , Shrestha, S. , & Chi, H. (2010). The S1P(1)‐mTOR axis directs the reciprocal differentiation of T(H)1 and T(reg) cells. Nature Immunology, 11(11), 1047–1056. 10.1038/ni.1939 20852647 PMC2958252

[brb32965-bib-0025] Mahdi‐Rogers, M. , Brassington, R. , Gunn, A. A. , van Doorn, P. A. , & Hughes, R. A. (2017). Immunomodulatory treatment other than corticosteroids, immunoglobulin and plasma exchange for chronic inflammatory demyelinating polyradiculoneuropathy. Cochrane Database of Systematic Reviews, 5(5), Cd003280. 10.1002/14651858.CD003280.pub5 28481421 PMC6481566

[brb32965-bib-0026] McGinley, M. P. , & Cohen, J. A. (2021). Sphingosine 1‐phosphate receptor modulators in multiple sclerosis and other conditions. Lancet, 398(10306), 1184–1194. 10.1016/s0140-6736(21)00244-0 34175020

[brb32965-bib-0027] Merkies, I. S. J. , van Schaik, I. N. , Léger, J. M. , Bril, V. , van Geloven, N. , Hartung, H. P. , & Mielke, O. (2019). Efficacy and safety of IVIG in CIDP: Combined data of the PRIMA and PATH studies. Journal of the Peripheral Nervous System, 24(1), 48–55. 10.1111/jns.12302 30672091 PMC6594229

[brb32965-bib-0028] Mohammad Jafari, R. , Shayesteh, S. , Ala, M. , Yousefi‐Manesh, H. , Rashidian, A. , Hashemian, S. M. , & Dehpour, A. R. (2021). Dapsone ameliorates colitis through TLR4/NF‐kB pathway in TNBS induced colitis model in rat. Archives of Medical Research, 52(6), 595–602. 10.1016/j.arcmed.2021.03.005 33814208

[brb32965-bib-0029] Müller, J. , von Bernstorff, W. , Heidecke, C. D. , & Schulze, T. (2017). Differential S1P receptor profiles on M1‐ and M2‐polarized macrophages affect macrophage cytokine production and migration. BioMed Research International, 2017, 7584621. 10.1155/2017/7584621 28367448 PMC5358463

[brb32965-bib-0030] Oaklander, A. L. , Lunn, M. P. , Hughes, R. A. , van Schaik, I. N. , Frost, C. , & Chalk, C. H. (2017). Treatments for chronic inflammatory demyelinating polyradiculoneuropathy (CIDP): An overview of systematic reviews. Cochrane Database of Systematic Reviews, 1(1), Cd010369. 10.1002/14651858.CD010369.pub2 28084646 PMC5468847

[brb32965-bib-0031] Rolland, W. B. , Lekic, T. , Krafft, P. R. , Hasegawa, Y. , Altay, O. , Hartman, R. , & Zhang, J. H. (2013). Fingolimod reduces cerebral lymphocyte infiltration in experimental models of rodent intracerebral hemorrhage. Experimental Neurology, 241, 45–55. 10.1016/j.expneurol.2012.12.009 23261767 PMC3570752

[brb32965-bib-0032] Shi, D. , Tian, T. , Yao, S. , Cao, K. , Zhu, X. , Zhang, M. , & Zhou, H. (2018). FTY720 attenuates behavioral deficits in a murine model of systemic lupus erythematosus. Brain, Behavior, and Immunity, 70, 293–304. 10.1016/j.bbi.2018.03.009 29548997

[brb32965-bib-0033] Skerjanec, A. , Tedesco, H. , Neumayer, H. H. , Cole, E. , Budde, K. , Hsu, C. H. , & Schmouder, R. (2005). FTY720, a novel immunomodulator in de novo kidney transplant patients: Pharmacokinetics and exposure‐response relationship. Journal of Clinical Pharmacology, 45(11), 1268–1278. 10.1177/0091270005279799 16239360

[brb32965-bib-0034] Tsuji, T. , Inoue, M. , Yoshida, Y. , Fujita, T. , Kaino, Y. , & Kohno, T. (2012). Therapeutic approach for type 1 diabetes mellitus using the novel immunomodulator FTY720 (fingolimod) in combination with once‐daily injection of insulin glargine in non‐obese diabetic mice. Journal of Diabetes Investigation, 3(2), 132–137. 10.1111/j.2040-1124.2011.00160.x 24843556 PMC4020730

[brb32965-bib-0035] White, C. , Alshaker, H. , Cooper, C. , Winkler, M. , & Pchejetski, D. (2016). The emerging role of FTY720 (Fingolimod) in cancer treatment. Oncotarget, 7(17), 23106–23127. doi:10.18632/oncotarget.7145 27036015 PMC5029614

[brb32965-bib-0036] Wu, X. , Xue, T. , Wang, Z. , Chen, Z. , Zhang, X. , Zhang, W. , & Wang, Z. (2021). Different doses of fingolimod in relapsing‐remitting multiple sclerosis: A systematic review and meta‐analysis of randomized controlled trials. Frontiers in Pharmacology, 12, 621856. 10.3389/fphar.2021.621856 34079453 PMC8165387

[brb32965-bib-0037] Yang, T. , Zha, Z. , Yang, X. , Kang, Y. , Wang, X. , Tong, Y. , Zhao, X. , Wang, L. , & Fan, Y. (2021). Neuroprotective effects of fingolimod supplement on the retina and optic nerve in the mouse model of experimental autoimmune encephalomyelitis. Frontiers in Neuroscience, 15, 663541. 10.3389/fnins.2021.663541 33981197 PMC8107225

[brb32965-bib-0038] Ye, F. , Xu, Y. , Lin, F. , & Zheng, Z. (2021). TNF‐α suppresses SHOX2 expression via NF‐κB signaling pathway and promotes intervertebral disc degeneration and related pain in a rat model. Journal of Orthopaedic Research, 39(8), 1745–1754. 10.1002/jor.24832 32816304

[brb32965-bib-0039] Zhang, F. , Yang, J. , Jiang, H. , & Han, S. (2014). An ανβ3 integrin‐binding peptide ameliorates symptoms of chronic progressive experimental autoimmune encephalomyelitis by alleviating neuroinflammatory responses in mice. Journal of Neuroimmune Pharmacology, 9(3), 399–412. 10.1007/s11481-014-9532-6 24577603

[brb32965-bib-0040] Zhang, L. , Sui, R. , & Zhang, L. (2022). Fingolimod protects against cerebral ischemia reperfusion injury in rats by reducing inflammatory cytokines and inhibiting the activation of p38 MAPK and NF‐κB signaling pathways. Neuroscience Letters, 771, 136413. 10.1016/j.neulet.2021.136413 34942319

[brb32965-bib-0041] Zhang, Z. , Zhang, Z. Y. , Fauser, U. , & Schluesener, H. J. (2008). FTY720 ameliorates experimental autoimmune neuritis by inhibition of lymphocyte and monocyte infiltration into peripheral nerves. Experimental Neurology, 210(2), 681–690. 10.1016/j.expneurol.2007.12.025 18261728

[brb32965-bib-0042] Zhao, S. , Adebiyi, M. G. , Zhang, Y. , Couturier, J. P. , Fan, X. , Zhang, H. , & Xia, Y. (2018). Sphingosine‐1‐phosphate receptor 1 mediates elevated IL‐6 signaling to promote chronic inflammation and multitissue damage in sickle cell disease. FASEB Journal, 32(5), 2855–2865. 10.1096/fj.201600788RR 29401601 PMC5901384

[brb32965-bib-0043] Zhu, Z. , Fu, Y. , Tian, D. , Sun, N. , Han, W. , Chang, G. , & Shi, F. D. (2015). Combination of the immune modulator fingolimod with alteplase in acute ischemic stroke: A pilot trial. Circulation, 132(12), 1104–1112. 10.1161/circulationaha.115.016371 26202811 PMC4580515

